# Transcriptomic analysis of *Rhodococcus opacus* R7 grown on polyethylene by RNA-seq

**DOI:** 10.1038/s41598-021-00525-x

**Published:** 2021-10-29

**Authors:** Jessica Zampolli, Alessandro Orro, Andrea Manconi, Diletta Ami, Antonino Natalello, Patrizia Di Gennaro

**Affiliations:** 1 Department of Biotechnology and Biosciences, University of Milano-Bicocca, Piazza della Scienza 2, 20126 Milan, Italy; 2 Institute of Biomedical Technologies, National Research Council, CNR, via Fratelli Cervi 19, Segrate, 20133 Milan, Italy

**Keywords:** Environmental microbiology, Gene expression, Genomics, Microbial genetics

## Abstract

Plastic waste management has become a global issue. Polyethylene (PE) is the most abundant synthetic plastic worldwide, and one of the most resistant to biodegradation. Indeed, few bacteria can degrade polyethylene. In this paper, the transcriptomic analysis unveiled for the first time *Rhodococcus opacus* R7 complex genetic system based on diverse oxidoreductases for polyethylene biodegradation. The RNA-seq allowed uncovering genes putatively involved in the first step of oxidation. In-depth investigations through preliminary bioinformatic analyses and enzymatic assays on the supernatant of R7 grown in the presence of PE confirmed the activation of genes encoding laccase-like enzymes. Moreover, the transcriptomic data allowed identifying candidate genes for the further steps of short aliphatic chain oxidation including *alkB* gene encoding an alkane monooxygenase, *cyp450* gene encoding cytochrome P450 hydroxylase, and genes encoding membrane transporters. The PE biodegradative system was also validated by FTIR analysis on R7 cells grown on polyethylene.

## Introduction

Plastics are an extensive group of synthetic polymers of fossil origin (i.e., crude oil and gas) or renewable (i.e., sugar cane, starch, and vegetable oils), or even mineral base (salt). In the last two centuries, plastic played a revolutionary role for its versatile properties, including convenience, non-degradability, durability, and low cost replacing other natural materials for packaging, transportation, storage, and garbage^1^. In 2018 plastics production almost reached 62 million tonnes only in Europe, and packaging by far represents the largest end-use markets^2^.

The most used synthetic polymer is polyethylene (PE) for its chemical–physical and mechanical properties such as high hydrophobicity, chemical resistance, electrical isolation, high breaking strength, high stability against deterioration, and low production costs^3^. In general, the Global Plastic industry is growing at a rate of 4.8% annually, significantly impacting the growth of the polyethylene market whose value is expected to rise to 215 billion U.S. dollars by 2024^4^. Different types of PE can be distinguished based on their density, the degree of branching, and the availability of functional groups on the surface: Low-Density Polyethylene (LDPE), High-Density Polyethylene (HDPE), Linear Low-Density Polyethylene (LLDPE), and Cross-Linked Polyethylene (XLPE)^5^.

Although the properties of PE are favorable for its long lifetime and durability, the same characteristic can lead to long-term impacts and risks as a major source of pollution for the environment. Indeed, the main issue is represented by the management of the PE end of life and waste disposal that still need to be addressed^6,7^. PE has always been considered as a chemically and biologically inert polymer with a degradation yield (mechanical or biodegradation) that can occur to a certain extent. The European Directive (UNI EN 13432:2002, 2002) defines biodegradable if more than 90% of the initial compound can be transformed into biomass, water, and CO_2_ within 6 months. Rather, PE deterioration requires a prolonged period under environmental conditions and some research studies reported microbial PE-degradative capacity^5,8,9^. Moreover, Albertsson and coworkers^10^ suggested that a synergistic effect between photooxidative degradation and biodegradation can facilitate the PE degradation: UV light and/or oxidizing agents begin the degradation, and after carbonyl group formation, microorganisms degrade the shorter segments starting from the oxidized PE chains forming carbon dioxide and water as end products. Indeed, different reports showed bacterial biodegradation of PE but only after the polymer was treated with acid^11^ or subjected to photooxidation or high temperatures beforehand^12–14^.

PE mineralization is a complex process that comprises the first oxidation of the hydrocarbon chain generating shorter aliphatic fragments and a consecutive degradation of the generated short alkanes^15^. PE biodegradability is inverse to its molecular weight: higher is the molecular weight, PE is more resistant to biodegradation whereas the higher number of branched molecules of LDPE can improve biodegradation^16^.

Proteobacteria and Firmicutes are two of the most common phyla of plastic degraders and more recently Actinobacteria demonstrated to have an important role in PE-contaminated soils^9^. Up to now, only 20 different bacterial genera are known to degrade diverse types of PE, including Gram-negative and Gram-positives bacteria belonging to *Pseudomonas*, *Ralstonia*, *Stenotrophomonas*, *Klebsiella*, *Acinetobacter*, *Staphylococcus*, *Streptococcus*, *Streptomyces*, *Micrococcus*, *Nocardia, Brevibacillus*, and *Bacillus* genera^1,5,17^. Among the others, few bacteria belonging to *Rhodococcus* genus possess the ability to oxidize and degrade PE. Microorganisms belonging to this genus are usually characterized by a versatile metabolism vs different organic contaminants, metals, and drugs and a unique adaptation to fluctuating and harsh environmental circumstances^14,15,18,19^. Moreover, it was demonstrated that only *Rhodococcus ruber* strain C208 was able to mineralize PE with and without prior abiotic factors, and RNA-seq analysis showed the induction of the pathways related to alkane degradation (*alkB* genes) and β-oxidation of fatty acids.

The involvement of the alkane degradation system was also demonstrated for *Pseudomonas aeruginosa* E7 and other *Pseudomonas* strains^20,21^. The AlkB engaging is likely explicable with the presence in PE samples or the formation of short aliphatic fragments, which could be recognized as the natural substrates/inducers. However, the complex mechanism of PE degradation requires prime oxidation to generate shorter aliphatic molecules as the first step^15^. In this perspective, Santo and coworkers^22^ reported a laccase-like multi-copper oxidase (LMCO) isolated from *R*. *ruber* C208 that has been proved to be able, when overproduced, to induce a reduction of the PE molecular weight. Nonetheless, transcriptomic analysis of C208 after growth on PE did not show any up-regulated laccases that are preferentially induced by lignin or aromatic compounds related to lignin or by metals, such as Cu^2+^.

In order to unravel the complex degradative system behind PE mineralization and identify the genetic determinants involved in this metabolism, a combination of genome-level techniques can help; thus, bacteria belonging to *Rhodococcus* genus can represent the perfect candidate for digging deeper.

In this paper, we report the transcriptomic analysis of *Rhodococcus opacus* strain R7 grown on PE to elucidate the biodegradative mechanism of polyethylene. RNA-seq allowed recovering genes putatively involved in the first step of oxidation. In-depth investigations through preliminary bioinformatic analyses and enzymatic assays on the supernatant of R7 grown in the presence of PE confirmed the activation of genes encoding laccase-like enzymes. Moreover, the transcriptomic data allowed identifying candidate genes for the further steps of short aliphatic chain oxidation including *alkB* gene encoding an alkane monooxygenase, *cyp450* gene encoding cytochrome P450 hydroxylase, and genes encoding membrane transporters. In addition, the PE biodegradative system was also validated by FTIR analysis on *R*. *opacus* R7 cells grown on polyethylene.

## Material and methods

### Bacterial strain and growth conditions

*R. opacus* strain R7 (CIP identification number 107348, deposited to the Institute Pasteur Collection)^23,24^ isolated from PAH contaminated soil, was generally cultivated in M9 mineral medium^25^ supplemented with 20 mM malate. Rapid screening growth assay was performed inoculating *R. opacus* R7 on M9 mineral medium agar plates supplemented with 1.2% polyethylene powder (Sigma-Aldrich 427772, Italy) in 1% Tween80 after vortexing for 1 min and then incubating at 30 °C for 48 h.

The growth assay was performed with a two-step inoculum: the R7 malate overnight culture was washed in mineral medium and inoculated in fresh M9 mineral medium supplemented with polyethylene powder (1.2%) as only carbon and energy source. After 48 h of growth, the PE culture was renovated in fresh M9 mineral medium supplemented with 1.2% PE powder. The inoculum was incubated at 30 °C under shaking (120 rpm) up to 144 h. The cultures were harvested at the late-exponential growth phase (optical density at 600 nm of 0.5 ± 0.05).

The R7 growth was evaluated also with the colony-forming unit counting method by serially diluting 0.1 mL of bacterial cultures in a solution of M9 mineral medium and plating on Luria–Bertani (LB) agar medium. Plates were incubated at 30 °C for 48 h. Data reported the mean of three biological replicates with standard deviation.

R7 culture in mineral medium M9 with 20 mM malate grown overnight at 30 °C (120 rpm) represented the reference condition. All conditions were performed in triplicates.

### Illumina high-throughput transcriptome sequencing

#### RNA extraction

Total RNA was isolated from *R. opacus* R7 cells (up to late-exponential phase) grown in 300 mL or 100 mL of M9 mineral medium supplemented with PE or malate, respectively.

Each growth condition was established in triplicated and from each culture the total RNA was extracted according to the manufacturer’s instructions of RNA Power Soil Total RNA Isolation Kit (Qiagen Italia, Italy) modifying the purification step that was performed by adding 3.5 mL of chloroform, mixing gently, centrifuging at 2500×*g* for 10 min, and transferring the upper phase to a clean tube. Then, the RNA was dissolved in 50 μL of RNase-free water. RNA concentration was evaluated with 6000 Nano kit (Agilent, Italy). For each sample, RNA quality was measured using Bioanalyzer Agilent 2100 supported with RNA 6000 Pico Agilent chip (Agilent, Italy). RNA quality check showed an integrity number (RNI value) major of 7.5 for all the RNA samples.

#### RNA sequencing

The preparation of the sequencing library and the selective rRNA depletion to enrich the samples of mRNA transcripts were performed using Universal Prokaryotic RNA-Seq (Nugen, USA). The RNA sequencing of the samples was performed by Illumina HiSeq platform (Biodiversa, Italy) generating about 110 million and 113 million pairs of raw reads (forward and reverse strands) for the three malate samples and the three PE samples, respectively. The quality of raw sequencing data was checked with Trim Galore (version 0.4.4) (URL: http://www.bioinformatics.babraham.ac.uk/projects/trim_galore) to trim the sequencing reads^26^, while GPU-Dup Removal was applied to identify and remove duplicates^27^. The quality control resulted in about 92 million and 60 million reads for malate and PE samples, respectively. The sequences of RNA-seq are submitted to ENA with the following accession number PRJEB45685.

#### RNA-seq data analysis and differential gene expression

A reference-based strategy was used to assemble the transcriptome exploiting the genome sequences of *R. opacus* R7 previously obtained^28^. RNA-seq reads were mapped on R7 genome using TopHat aligner^29^ and they were separately processed and assembled by the Cufflinks default pipeline^30^ into a set of transcript fragments. The assembling process generated a total of 9679 transcripts with an overall read mapping rate of 54% and 85% for PE and malate samples, respectively. Then, the transcripts were merged in a format suitable for Cuffdiff software^31^ in order to obtain a set of loci with statistically different expression levels using default parameters. The final result was a table reporting the loci information (identifier, scaffold, start and stop location), the expression values (proportional to the number of reads mapping the locus), and the significance level (p-value) (Table S1). Lastly, the loci were ranked, filtered by p-value, and annotated by assigning the annotation of the corresponding Open Reading Frame (ORF). In addition, the expression values were adjusted by substituting the zero value of the expression with the minimum expression value of the dataset (excluding zero value).

#### Gene cluster annotation

A total of 9679 ORFs were predicted by Glimmer^32^ and annotated with Rapid Annotation using Subsystem Technology (RAST)^33^ resulting in function and subsystem classification. 9679 functionally annotated ORFs were grouped in subsystems that represent biological functional roles.

The differentially expressed genes (DEGs) were also manually analyzed using BLAST^34^ with e-value < 10 and Clustal Omega^35^ against reference sequences from the UniProt database^36^ and literature searches.

Data resources used in gene cluster annotation include: KEGG^37^ and GO^38^. For each KEGG and GO term, the count of genes has been computed and compared between the two growth conditions.

### Quantitative real-time PCR (RT-qPCR)

The transcriptional induction of the *R*. *opacus* R7 genes potentially encoding enzymes involved in PE degradation was evaluated by quantitative reverse transcription (RT-) qPCR experiments. The amplified genes encode multicopper oxidases, LMCO1, 2, 3, and *alkB* gene and 16S rRNA used as a reference.

The reverse transcription of the total RNA was performed with iScriptcDNA Synthesis kit (BIO-RAD, Italy) to obtain 200 ng cDNA with the following thermocycling conditions: 5 min at 25 °C followed by 1 h at 42 °C and then 5 min at 85 °C.

The amplification of the cDNA obtained was carried out using the StepOnePlus Real-Time PCR System (Applied Biosystem, Italy) in 10-µL qPCR volume containing 4.4 µL of the reverse-transcribed RNA samples, 5 µL of PowerUp SYBR Green Master Mix (Applied Biosystem, Thermo Scientific, Italy), and 300 nM of each primer listed in Table S2. The temperature program was as follows: 30 s at 95 °C, followed by 40 cycles of 5 s at 95 °C, 10 s at 60 °C and 45 s at 72 °C and one cycle of 15 s at 95 °C, 1 min at 60 °C and 15 s at 60 °C.

The expression of 16S rRNA was used as a reference^39^ to normalize the tested gene expression according to the ΔΔCt method^40^. The malate growth condition was used as a reference in order to determine the relative abundance of target transcripts. Data are expressed as mean ± standard deviation derived from at least three independent experiments.

In order to exclude DNA contamination, negative controls were performed by omitting the reverse transcriptase in RT-PCR experiments, which were conducted with the same temperature program and the same primer sets for 35 cycles of amplification.

### Bioinformatics analysis for the enzyme characterization

Based on RNA-seq data, we considered the aa sequences of multicopper oxidases induced after growth on PE and predicted with GO annotation and KEGG as putative laccase proteins (E.C. 1.10.3.2). Moreover, they were analyzed with EFICAz program^41^ to verify their enzymatic classification (EC number) and compared with PROSITE^42^ patterns to identify the conserved specific laccase domains (PDOC00076) corresponding to:

$$\begin{gathered} {\text{G-x-}}[{\text{FYW}}]{\text{-x-}}[{\text{LIVMFYW}}]{\text{-x-}}[{\text{CST}}]{\text{-x-}}\{ {\text{PR}}\} {-}\{ {\text{K}}\} {\text{-x}}(2){-}\{ {\text{S}}\} {\text{-x-}}\{ {\text{LFH}}\} {\text{-G-}}[{\text{LM}}]{\text{-x}}(3){-}[{\text{LIVMFYW}}]\;\;{\text{and}} \hfill \\ {\text{H-C-H-x}}(3){\text{-H-x}}(3){-}[{\text{AG}}]{-}[{\text{LM}}]. \hfill \\ \end{gathered}$$

Moreover, laccase aa sequences were retrieved from Protein Data Bank^43^ and aligned against *R. opacus* R7 putative laccases (UniProt ID: I6WZK7 and Q02219) to find the 3D structures with high similarity (more than 25% of percent identity).

In addition, the location of the correspondent protein was investigated predicting the presence of Twin-arginine signal peptide (Tat) by performing analyses through TATFIND v.1.4^44^ (http://signalfind.org/tatfind.html), TatP v.1.0^45^, SignalP-5.0^46^ (http://www.cbs.dtu.dk/services/SignalP-5.0/index.php) and comparing their results.

Moreover, in order to evaluate the gene candidates retrieved from the RNA-seq analysis BLASTx tool of NCBI pipeline^34^, Clustal Omega^35^, and KEGG^37^ were used to determine and compare amino acid sequence homology and to make manual curation.

### Enzymatic assay

After R7 strain growth on PE or malate, a cell-free fraction (CFS) was obtained by centrifugation at 7000 rpm. Residual PE was removed by filtration with 0.45 µm filters (Millipore). The supernatant was lyophilized and the product originating from 100 mL of R7 culture was redissolved in 2 mL 50 mM potassium phosphate buffer (pH 7). This resuspension was dialyzed overnight against 400 mL of 0.2 mM potassium phosphate buffer (pH 7).

The specific activity of the lyophilized and dialyzed CFS fraction was determined in 50 mM potassium phosphate buffer (pH 7). Laccase specific activity was defined as the amount of protein required to oxidize 30 mM of 2,6-dimethoxyphenol (DMP) by increasing absorbance at 470 nm during 5-min intervals, for up to 60 min. Laccase activity tests were carried out in a UV/visible spectrophotometer (Ultraspec 2100 *pro*, Amersham Biosciences). The specific activity was calculated as U mg^−1^. The total protein concentration of the lyophilized and dialyzed CFS was assessed using the method of Bradford^47^ using Coomassie brilliant blue with bovine serum albumin as a standard. Protein concentrations were calculated from the standard curve by 20 µg mL^−1^ bovine albumin serum.

The activity and stability of R7 CFS were compared to the commercial fungal *T*. *versicolor* laccase (≥ 0.5 U mg^−1^) (Sigma-Aldrich, Italy).

### Fourier transform infrared for the characterization of *R*. *opacus* R7 cells grown on polyethylene

*R*. *opacus* R7 cells grown on M9 mineral medium supplemented with 1.2% PE, 20 mM malate, or M9 mineral medium until the late-exponential phase were washed four times in physiological solution (0.9% NaCl w/v in sterile Milli-Q water) at 8000 rpm for 5 min.

After resuspension of cell pellets in few microliters of 0.9% NaCl, ~ 2 µL of the resulting solutions were deposited onto a BaF2 window and dried at room temperature for about 30 min to eliminate the excess water.

FTIR absorption spectra were acquired in transmission mode, between 4000 and 700 cm^−1^, by the infrared microscope, Varian 610-IR coupled to the Varian 670-IR FTIR spectrometer (Varian Australia Pty Ltd., Mulgrave, VIC, Australia), equipped with a mercury cadmium telluride nitrogen-cooled detector. The variable microscope aperture was adjusted to 100 µm × 100 µm. Measurements were performed at 2.0 cm^−1^ spectral resolution, 25 kHz scan speed, triangular apodization, and by the accumulation of 512 scan co-additions.

When necessary, the spectra were corrected for the residual water vapor and then normalized at the Amide I band area. The second derivative analysis was performed by the Savitzky–Golay method (3rd polynomial, 9 smoothing points), after a 13-point smoothing of the measured spectra, using the GRAMS/32 software (Galactic Industries Corporation, Salem, NH, USA).

For each sample, several measurements were performed by selecting different areas on the same sample (7–10 spectra for each condition in each experiment). Moreover, the reproducibility of the results was evaluated with three independent experiments.

## Results

### Growth of *R. opacus* R7 on polyethylene

**Figure 1:**
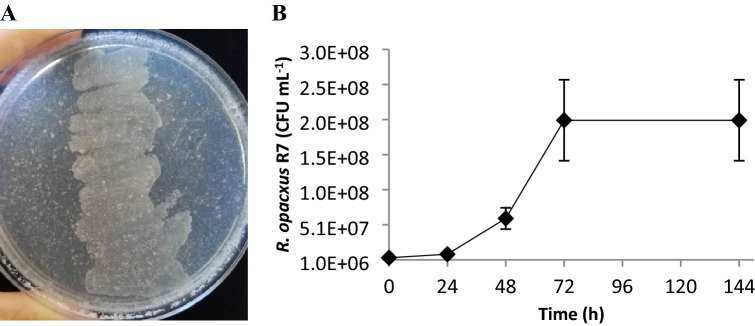
*R*. *opacus* R7 growth on polyethylene. (**A**) Shows preliminary growth assay performed inoculating *R. opacus* R7 on M9 mineral medium agar plates supplemented with 1.2% PE and 1% Tween80. (**B**) Shows R7 kinetic growth on M9 mineral medium supplemented with PE 1.2% as the only carbon and energy source.

*R. opacus* strain R7, which was selected for its ability to degrade numerous aliphatic, mono- and polycyclic aromatic hydrocarbons, and cyclo-carboxylic acids^24^, was preliminarily tested for the ability to grow on PE by a plate agar assay dissolving the PE powder in 1% Tween80. As shown in Fig. 1A, in the area of R7 cell deposition, the strain was able to grow in the presence of PE as the only carbon and energy source.

Consequently, a growth assay of *R. opacus* strain R7 was carried out in liquid M9 mineral medium culture supplemented with 1.2% PE up to 144 h. R7 ability to degrade PE was evaluated by the colony-forming unit method over time (Fig. 1B). The result showed that the amount of R7 cells increased almost two orders of magnitude in 48 h and the maximum growth resulted in a total cell amount of around 2 × 10^8^ CFU mL^−1^ at 72 h.

Moreover, the modifications of content and structure of whole R7 cell were evaluated in the presence of polyethylene compared to malate condition by FTIR microspectroscopy (as reported in the paragraph FTIR characterization of *R. opacus* R7 intact cell grown on polyethylene).

### Overview of transcriptomic data from *R. opacus* R7 grown on polyethylene

The biodegradative mechanism of polyethylene by *R. opacus* R7 was studied through a transcriptomic approach performed after 72 h of R7 growth on PE by RNA-seq technology. R7 grown in the presence of malate was used as a reference for the transcriptomic analysis.

A reference-based strategy was used to assemble the transcriptomes and it produced a total of 9679 transcripts with an overall read mapping rate of 54% and 85% for PE and malate samples, respectively. Quality control produced a total of 92 and 60 million reads for PE and malate conditions, respectively. The differential gene expression was obtained by calculating the log_2_(fold_change) that from now is named LogFC, for the two different growth conditions.

Among the total number of R7 coding DNA sequences (CDSs), 97% were not showing appreciable changes of gene expression under both conditions, while considering p-value < 0.05, 130 (1.3%) and 196 (2%) genes were up- and down-regulated, respectively. The genes with a LogFC > 2 (and p-value < 0.05) were only 2.6% for both conditions. Overall, the up-regulated genes (p-value < 0.05) showed a LogFC varying from 2 to 21.6 while the down-regulated genes from − 2 to − 20 (Table S1).

Abnormal LogFC values were shown by 19 differentially expressed genes (DEGs) due to the switch-off/-on effect^48^, thus they were corrected with a min-value approximation resulting in an approximated LogFC varying from − 20 to 21. Among the 19 DEGs with corrected LogFC, 5 were up-regulated genes, 14 were down-regulated genes, and 11 genes were preliminary annotated as hypothetical proteins (Table S1).

The DEGs were variously distributed on *R*. *opacus* R7 genome that is constituted by one chromosome and five plasmids (pPDG1, pPDG2, pPDG3, pPDG4, and pPDG5): the highest number of up-regulated genes are located on the chromosome and pPDG1 and pPDG2 plasmids with 87% and 6% DEGs with respect to the total number of up-regulated genes, respectively. Instead, no up-regulated gene was reported on pPDG3, pPDG4, and pPDG5 plasmids. The down-regulated genes were predominantly located on R7 chromosome (83%) and, 3%, 4%, 10%, and 1% DEGs with respect to the total number of down-regulated genes were present on pPDG1, pPDG2, pPDG3, and pPDG4 plasmids, respectively. Notably, no down-regulated genes were recorded on the pPDG5 plasmid.

After Glimmer prediction, 9679 ORFs were functionally annotated with RAST. Afterward, the transcripts were further classified by GO and KEGG database using BLAST search against UniProt. In total, the annotation process allowed the classification of transcripts by RAST (9664 total number), GO (8991 total number), and KEGG (3416 total number) databases.

**Figure 2:**
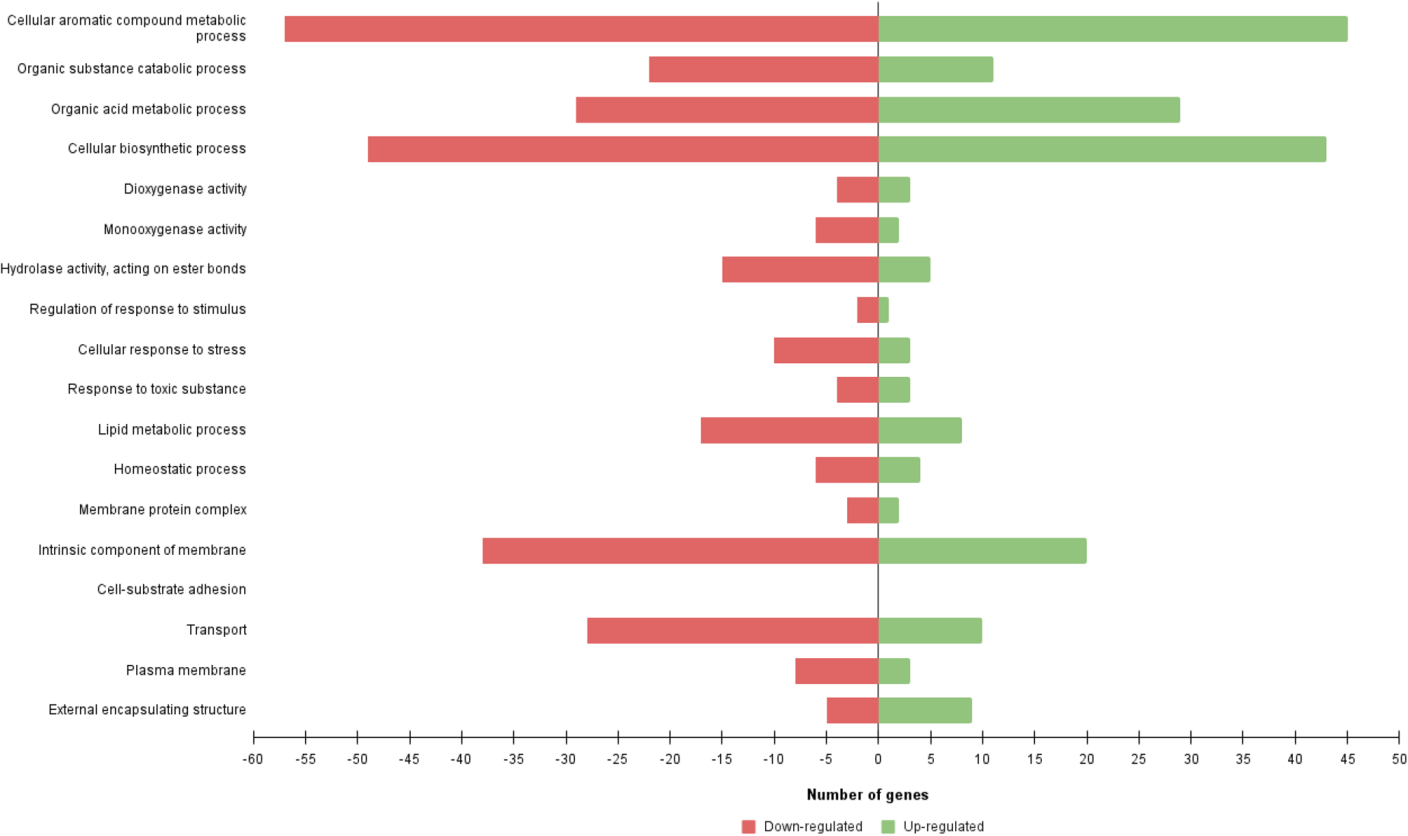
Distribution of *R*. *opacus* R7 DEGs respect with polyethylene and malate growth conditions according to physiological categories. Barplot shows the number of up- and down-regulated genes associated with GO categories such as cellular aromatic compound metabolic processes, organic substance catabolic processes, organic acid metabolic processes, cellular biosynthetic processes, dioxygenase activity, monooxygenase activity, hydrolase activity, acting on ester bonds, regulation of response to stimulus, cellular response to stress, response to toxic substance, lipid metabolic processes, homeostatic processes, membrane protein complex, intrinsic component of membrane, cell-substrate adhesion, transport, plasma membrane, external encapsulating structure.

Figure 2 represents the number of R7 DEGs associated with the main GO categories, such as organic substance catabolic process, dioxygenase, monooxygenase, hydrolase activity, and transport. Out of 301 assigned DEGs to GO categories 271 were assigned to “molecular functions”, 266 to “biological processes”, and 178 to “cellular components”. The highest percentage of transcripts were classified under the following GO categories: 74% of DEGs under GO annotation as “metabolic processes”, 68% as “catalytic activity”, and 66% as “cellular processes”.

**Figure 3:**
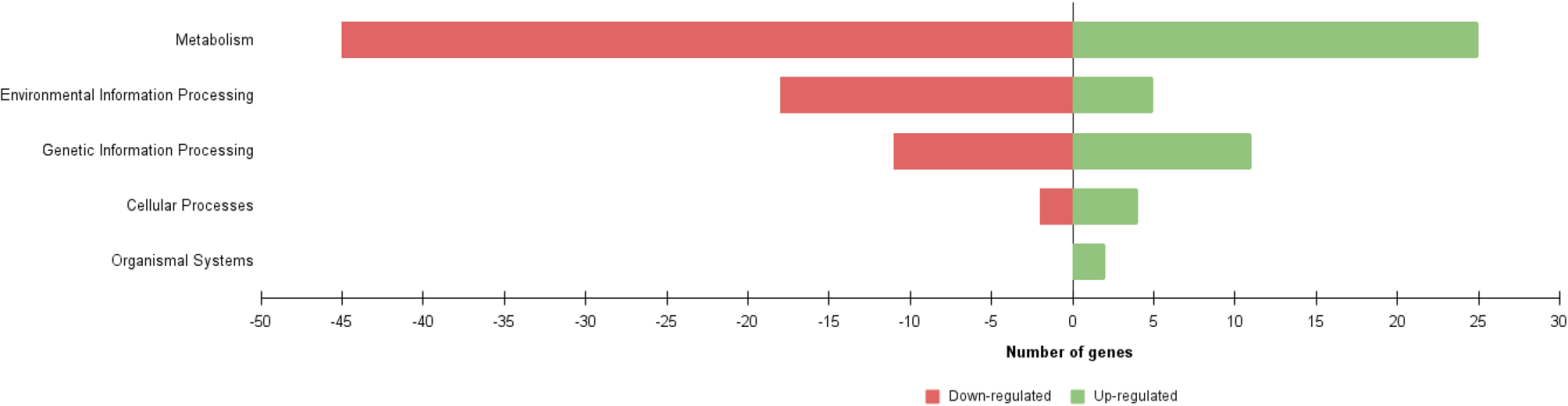
Number of up- and down-regulated genes of *R*. *opacus* R7 in response to polyethylene and malate according to KEGG identifiers. Distribution of the main KEGG classes including metabolism, environmental information processing, genetic information processing, cellular processes, and organismal systems.

The transcripts were also classified by KEGG identifiers exhibiting 384 DEGs associated with 343 pathways. Among the KEGG-annotated sequences, 70 were grouped under the “metabolism” category (63% of total DEGs), 23 under “environmental information processing” (21%), 22 under “genetic information processing” (20%), 6 under “cellular processes” identifier (5%), and 2 under “organismal systems” (3%) (Fig. 3). More in detail, 13 into “biosynthesis of various secondary metabolites”, 13 into “microbial metabolism in diverse environments”, 6 into “carbon metabolism", and 1 into “ABC transporters”.

Overall, the transcriptome showed DEGs associated with oxidoreductase activity, hydrocarbon catabolic processes, central metabolism, transporters, and membrane activity. On the other hand, the analyses also showed that 15% of the positively regulated genes and 18% of the negatively regulated genes were preliminary annotated as hypothetical proteins (HP) with unknown functions with respect to total DEGs (Table S1).

Based on the classification of GO and KEGG databases, differentially regulated genes connected to stress and translation were also identified. Specifically, eleven differentially regulated genes related to stress including among the other genes putatively encoding a xylose repressor XylR (ROK family), a flavohemoprotein (hemoglobin-like protein) (flavohemoglobin) (nitric oxide dioxygenase) (EC 1.14.12.17), a thiol peroxidase, Bcp-type (EC 1.11.1.15). a l-ectoine synthase (EC 4.2.1.–), a possible universal stress protein, a stress responsive A/B Barrel Domain superfamily protein, and a heat shock protein 60 family chaperone GroEL were detected in the RNA-seq. On the other hand, few genes related to stress response were down-regulated including genes encoding proteins for DNA recombination and repair, l-proline biosynthetic process and response to copper ion. Moreover, nine up-regulated genes showed a GO annotation related to translation including six annotated as hypothetical proteins, and a collagen triple helix repeat, a TsaC protein (YrdC domain) required for threonylcarbamoyladenosine t(6)A37 modification in tRNA, and a LSU ribosomal protein L11p (L12e). On the other hand, two down-regulated genes annotated as hypothetical proteins showed a GO annotation related to translation.

### Identification of specific pathways induced in *R*. *opacus* R7 grown on polyethylene

In order to identify the genetic determinants involved in polyethylene degradation by *R*. *opacus* strain R7, the transcriptomic data were explored searching for genes potentially involved in the biodegradative pathway, including genes encoding oxygenases and oxidases.

Accordingly, RNA-seq data highlighted a *R*. *opacus* R7 gene, LMCO1 (AII08809) that was described as a multicopper oxidase by the preliminary annotation and was up-regulated 19.5-fold. LMCO1 gene is located on the chromosome downstream a genome region comprising genes encoding a benzoate dioxygenase system and two-component monooxygenase [(4-hydroxyphenylacetate 3-monooxygenase (EC 1.14.13.3) and nitrilotriacetate monooxygenase component B (EC 1.14.13.–)] (PheA1).

Among R7 oxidases, the other two multicopper oxidases were considered although the RNA-seq analysis did not show evidence of their induction. They are located on pPDG3 plasmid: LMCO2 gene (AII11185) is located in a genome region including genes encoding (copper-) membrane transporters; LMCO3 gene (AII11221) is located just down-stream genes encoding copper resistance genes and up-stream carbonic anhydrase, and zinc-transporter genes.

**Figure 4:**
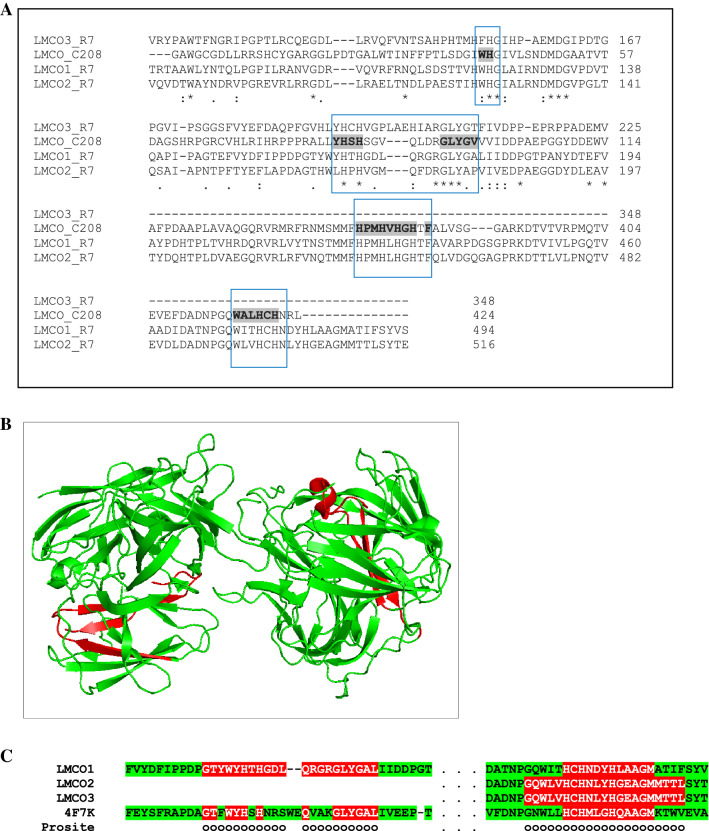
Bioinformatics analyses of amino acid sequences of *R. opacus* R7 LMCO gene products encoded by LMCO1, 2, and 3 DEGs. Multiple alignment of LMCO1, 2, and 3 amino acid sequences with respect to *R*. *ruber* C208 LMCO sequence evidencing putative copper-binding motifs generally associate to the class of laccases (rectangular area); bold grey amino acids correspond to the copper-binding motifs reported for C208 sequence^22^ (**A**). Protein structure of Lac15 from a marine microbial metagenome (PDB 4F7K) showing in red color the laccase domain regions (**B**). Multiple alignment of the conserved domain region of 4F7K sequence respect with LMCO1, 2, and 3 amino acid sequences according to EFICAz software and PROSITE (**C**).

In order to bioinformatically analyze the three R7 multicopper oxidases, LMCO aa sequences were compared to the laccase-like enzyme sequence identified in *R*. *ruber* C208 genome for its role in PE degradation. The alignments (performed on the NCBI pipeline) showed that the amino acidic identity of C208 laccase was 48% with respect to LMCO1, 53% with respect to LMCO2, and 23% with respect to LMCO3. Moreover, R7 LMCO aa sequences were aligned vs NCBI pipeline identifying all the copper-binding sites characteristic of laccase enzymes^49,50^ in two of the three R7 oxidases (LMCO1 and LMCO2), while only two copper-binding sites were identified in LMCO3 (Fig. 4A). R7 LMCO aa sequences were further characterized with EFICAz software and PROSITE pattern search that confirmed that two aa sequences were predicted as putative multicopper oxidases (LMCO1 and LMCO2) which also showed more than 25% of sequence identity with respect to a “Crystal structure of Lac15 from a marine microbial metagenome” (PDB structure 4F7K) as shown in Fig. 4B,C. The other aa sequence (LMCO3) does not have a specific known laccase domain but shows 57% of sequence identity with respect to the crystal structure of a reference multicopper oxidase (PDB structure: 3GDC).

In addition, R7 aa LMCO sequences were screened for signal amino acids (aas) for the translocation of folded proteins across lipid bilayers^51^. All three R7 sequences evidenced conserved twin-arginine (TAT) signal peptide motif consisting of (S/T)RRxFL (with x referring to a polar amino acid) and a hydrophobic region of an average length among 18 and 22 amino acids, located downstream TAT signal aas and up-stream the predicted cleavage peptides.

After the initial oxidative step of polyethylene through the secretion of extracellular oxidases, efficient mineralization of polyethylene would require additional steps, including (i) specific transport systems that can be hypothesized for short length-oxidized-PE fragments, (ii) additional cytoplasmic oxidations to finalize small PE fragments oxidation, and (iv) the subsequent entrance of these fragments into the beta-oxidation pathway.

Therefore, based on this hypothesis, 5 different transcripts (AII08802, AII09363, AII08803, AII03285, and AII08421) encoding oxygenases/hydroxylases were considered for their induction during growth on PE, showing a LogFC ranging from 4 to 19.4 (Table S1, Table S3). Among these up-regulated genes, three genes encode respectively a sarcosine oxidase delta subunit, a 2-polyprenylphenol hydroxylase, and related flavodoxin oxidoreductases, and a conserved hypothetical hydroxylase, putatively involved in R7 stress response; one benzoate dioxygenase gene probably activated for its role in the central metabolism, and only one gene coding for a putative cytochrome P450 hydroxylase (AII08421) (Table S3). AII08421 was similar to cytochrome P450 hydroxylase CYP124 of *Mycobacterium* spp. with an aa identity of 32% (PDB: 2WM4, 6T0F, and 6CVC). Moreover, amino acid sequence comparison between R7 P450 hydroxylase and CYP125 of *Rhodococcus erythropolis* PR4 (RER_33720) or *Rhodococcus jostii* RHA1 (WP_009477746) showed 31% and 29% identity, respectively^52^. Therefore, the preliminary bioinformatics analyses of AII08421 amino acid sequence revealed that it could be included in CYP124 or CYP125 families. Indeed, the analysis of CYP124A1 substrate specificity for *Mycobacterium* strains indicated that it catalyzes the terminal hydroxylation of methyl-branched hydrocarbon chains^53^; *cyp125* genes of *R. erythropolis* PR4 are activated in the presence of diesel oil, while CYP125 of *R. jostii* RHA1 is fundamental to initiate the catalysis of 3-hydroxysterols (cholesterol) via sterol side-chain degradation^54^. Overall, these preliminary suggestions indicated the possible involvement of R7 cytochrome P450 gene in the oxidation of internalized PE short chains.

Likewise, there is a redundancy of genes in R7 genome encoding other monooxygenases/hydroxylases. For example, we considered the alkane-1-monooxygenase encoding gene since its involvement in the degradation of medium-chain *n*-alkanes (C_12_) has been verified in the previous work^55^. R7 *alkB* (AII08632) is a unique gene in the genome and it is expressed during R7 growth on PE. AlkB is also reported to be involved in PE degradation since it is induced by medium-chain alkanes^15,20,21^.

Consistent with this hypothesis, diverse R7 genes putatively encoding membrane protein (AII10792), integral membrane protein (AII10609), and putative ATP/GTP-binding integral membrane protein (AII04246) appeared up-regulated during the R7 PE degradative process with LogFC 5.5, 3, and 2, respectively. Amino acid sequence alignment of these three membrane proteins against NCBI pipeline showed 67%, 84%, and 88% identity with DUF3556 domain-containing protein, DUF475 domain-containing protein, and ATPase. In addition, other gene candidates were retrieved from genome analysis such as genes encoding an ABC transporter ATP-binding protein (AII09203), a protein-export membrane protein SecD (TC 3.A.5.1.1) (AII04221), and other membrane proteins (AII06740 and AII07038) which could have a role in PE metabolism.

Finally, the PE mineralization can be achieved through the β-oxidation pathway. Indeed, R7 genes putatively involved in this pathway were up-regulated with LogFC varying between 18 and 4 under PE growth as observed by^15^. Indeed, the metabolism of short-medium chain alkanes usually proceeds through a (NAD(P)-dependent) alcohol dehydrogenase (AII08419), aldehyde dehydrogenase (EC 1.2.1.3) (AII08418), acetaldehyde dehydrogenase (EC 1.2.1.10) (AII03621), long-chain-fatty-acid-CoA ligase (EC 6.2.1.3) (similar to WP_128639699), 3-hydroxybutyryl-CoA dehydrogenase (EC 1.1.1.157)/3-hydroxyacyl-CoA dehydrogenase (EC 1.1.1.35) (AII05967).

### RT-qPCR analysis of selected *R*. *opacus* R7 genes from RNA-seq

**Figure 5:**
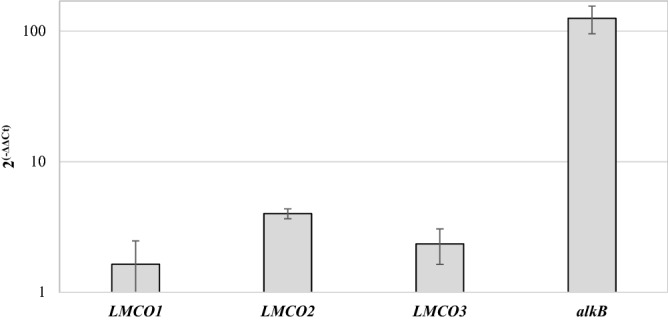
Expression levels of R7 selected genes for RT-qPCR analysis. The selected genes are *LMCO1*, *LMCO2*, *LMCO3*, and *alkB* encoding laccase-like multicopper oxidase 1, 2 and 3 and alkane monooxygenase, respectively.

RT-qPCR experiments were performed on cDNA deriving from R7 cell grown in the presence of PE or malate in order to validate the transcription levels of R7 candidate genes putatively involved in PE degradation. We selected genes encoding oxidases and oxygenases that could be good candidates for the two main oxidative steps of PE degradation: the first oxidation on the cellular membrane or in the extracellular environment and the cytoplasmic oxidation of PE when shorter fragments of polymer are internalized. Therefore, we selected *LMCO1* (AII08809), *LMCO2* (AII11185), *LMCO3* (AII11221) genes encoding multicopper oxidases, and *alkB* (AII08632) gene encoding an alkane-1-monooxygenase. Figure 5 shows the values of RT-qPCR activation obtained with the ΔΔCt method for each selected candidate gene.

The gene expression evaluated by RT-qPCR showed identical trends in terms of the direction of differential expression (up-regulation) for all the tested genes with respect to RNA-seq.

### Enzyme assay on *R*. *opacus* R7 supernatant

Based on RNA-seq data and preliminary bioinformatic analyses, we hypothesized that *R*. *opacus* R7 grown on PE produces an extracellular degradative enzyme system similar to laccase-like. Therefore, the laccase-like activity was monitored collecting the CFS at maximum growth level of *R*. *opacus* R7 on PE after 72 h. The enzymatic activity of R7 CFS reached a maximum of 10^–6^ U mg^−1^. Additionally, the corresponding CFS was concentrated 50 and 500 times without significant loss of activity.

Moreover, the laccase activity was assessed on CFS isolated from R7 culture on malate and it was equal to 6 × 10^–8^ U mg^−1^. Notably, the laccase-like activity evidenced in the supernatant without cells (CFS) of R7 grown on PE was higher with respect to the CFS obtained from R7 grown on malate. We can speculate that this laccase-like activity could correspond to the activity exerted by LMCO genes that were up-regulated in the transcriptomic analysis.

### FTIR characterization of *R*. *opacus* R7 intact cells grown on polyethylene

**Figure 6:**
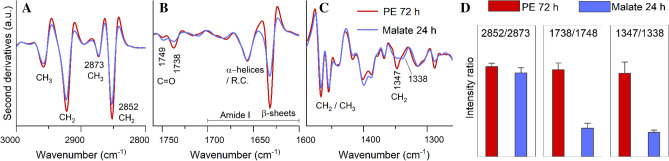
FTIR analysis of *R. opacus* R7 intact cells. The second derivatives of the mean FTIR absorption spectra of *R*. *opacus* R7 cells grown on PE for 72 h and malate for 24 h are reported in the spectral ranges: (**A**) 3000–2800 cm^−1^, (**B**) 1760–1600 cm^−1^, (**C**) 1500–1250 cm^−1^. The peak position and the assignment at selected vibrational modes are indicated. Panel (**D**) shows the intensity ratios between the reported peaks (2852 cm^−1^/2873 cm^−1^, 1738 cm^−1^/1748 cm^−1^, 1347 cm^−1^/1338 cm^−1^), taken in the second derivative spectra. The error bars represent the standard deviation from three independent experiments.

To disclose possible modifications in the content and the structure of the whole *R*. *opacus* R7 cell biomolecules induced by PE as the only carbon and energy source, compared to malate condition, FTIR microspectroscopy was employed. This vibrational approach enables to characterize not only isolated biomolecules but also complex biological systems, such as intact cells, providing a snapshot of the structure and content of the main molecules^56^. For sake of clarity, only the spectral bands displaying the most important spectral differences in the two growth conditions will be described. In particular, we analyzed the second derivative spectra in the spectral ranges where the functional groups of the main biomolecules absorb (Fig. 6A–C).

Between 3000–2800 cm^−1^ (Fig. 6A), the second derivative spectra are characterized by four well resolved bands assigned mainly to lipid hydrocarbon chain vibrational modes: ~ 2958 cm^−1^ (CH_3_ asymmetric stretch), ~ 2922 cm^−1^ (CH_2_ antisymmetric stretch), ~ 2873 cm^−1^ (CH_3_ symmetric stretch), and ~ 2852 cm^−1^ (CH_2_ symmetric stretch)^57^. In particular, the CH_2_ bands display a higher intensity in the PE cell spectrum compared to control cells (Fig. 6A,D). In the spectral range 1760–1600 cm^−1^, the second derivative spectra show two peaks mainly ascribable to carbonyl groups involved in ester bonds^56^, at ~ 1749 cm^−1^ and ~ 1738 cm^−1^, with a different intensity in the two conditions (Fig. 6B,D). In addition, also the Amide I band components, due to the C=O stretch of the peptide bond and giving information on protein secondary structures, are observed at ~ 1657 cm^−1^ (assigned to protein alpha helix/random coil structures), and at ~ 1632 cm^−1^ with higher intensity in PE cells (due to protein *beta*-sheets) (Fig. 6B)^56^.

Finally, in the spectral range 1500–1250 cm^−1^, absorption bands mainly assigned to hydrocarbon chain vibrational modes occur: the ~ 1467 cm^−1^ (deformation modes of CH_2_/CH_3_)^57^ that, in agreement with what found between 3000–2800 cm^−1^, is of higher intensity in PE cells compared to control; a complex absorption between ~ 1361 and 1331 cm^−1^, which is assigned to hydrocarbon chain CH_2_ wagging vibrations^57^. Here, in PE cells, the component at ~ 1347 cm^−1^ is at higher intensity compared to control where, instead, also a component at ~ 1338 cm^−1^, negligible in PE cells, is found (Fig. 6C,D).

Overall, the FTIR results indicate that *R*. *opacus* R7 growth on PE leads to spectral differences, compared to the growth on malate, involving mainly the response of lipids and proteins. These differences could reflect a different metabolism in the two cases.

## Discussion

The importance of addressing the issue of plastic disposal is nowadays of great relevance. Among the different kinds of plastic and plastic wastes, polyethylene has gained the center of attention due to its properties of non-degradability and durability^3^. Certainly, these features have ensured its large utilization and duration but on the other hand have posed the urgent problem of plastic waste disposal. In general, biodegradation is an eco-friendly approach to apply to different fractions of plastic wastes, thus more versatile with respect to chemical or mechanical processes.

Up to now, few bacterial genera have been characterized for the ability to degrade diverse types of PE^20,21^, at least partially, and only *Rhodococcus ruber* C208 has been investigated for the genetic determinants involved in this metabolism^15^.

Interestingly, *R*. *opacus* R7 is capable to grow in the presence of mineral medium supplemented with PE as the only carbon and energy source. R7 growth culture reached a high level of cell amount after 72 h with a growth profile similar to the one observed for *R*. *ruber* C208^15^. Although the induction of the pathways related to alkane degradation and β-oxidation of fatty acids have been identified in C208 grown on PE, the complex gene framework involved in PE biodegradation is still unclear and not completely explored. Therefore, the present study aims at in-depth reconstruction of *R*. *opacus* R7 gene framework for PE degradation through the RNA-seq approach evaluating the possible involvement of specific enzymatic systems. This work shows *R*. *opacus* R7 transcriptome analysis performed after three days of growth on PE to identify genes encoding candidate enzymes involved in PE degradation.

The RNA-seq approach allowed uncovering a total of 130 up-regulated genes (p-value < 0.05) of *R*. *opacus* R7. Newsworthy, these up-regulated genes were mostly located on the chromosome (87%). Although several transcripts were associated with hypothetical proteins (HP), the GO and KEGG analysis showed that several up-regulated transcripts were associated with oxidoreductase activity, hydrocarbon catabolic processes, central metabolism, transporters, and membrane activity; in addition, few DEGs related to stress or translation were also identified.

Considering oxidoreductase activity, specifically oxidative activity, R7 RNA-seq data together with RT-qPCR experiments highlighted three up-regulated genes annotated as multicopper oxidases (LMCO1, 2, 3). The predictive bioinformatics analyses indicated that these genes belong to “laccase-like multicopper oxidase” (LMCO) enzymatic class^49^. Moreover, the results of *R*. *opacus* R7 transcriptome are in line with the report of a laccase-like enzyme (EC 1.10.3.2) able to catalyze externally the biotic oxidation of PE and the suggestion that the same class of enzyme could be responsible for the first PE oxidation step before of smaller PE fragments are internalized in *Rhodococcus* cells^15,22^. The predictive bioinformatic analyses on R7 LMCO1, 2, and 3 gene sequences supported the idea of their involvement in PE metabolism since the correspondent amino acid sequences possess the main conserved pattern domains and the copper-binding motifs typical of enzymes possibly involved in PE degradation^1^.

In addition, enzymatic assays for laccase activity performed on R7 CFS collected after growth on PE added the credence that the detected laccase-like activity could correspond to the activity exerted by LMCO genes that were up-regulated in transcriptomic analysis. Moreover, the laccase-like activity detected in R7 CFS is comparable to the laccase activity of C208 strain without copper addition^22^.

The overall analysis of RNA-seq data showed also that other transcripts encoding oxygenases/oxidases were up-regulated and they could participate in further steps of intracellular PE-smaller fragment oxidation as reported by Yoon et al., Jeon and Kim, and Gravouil et al.^15,20,21^. Specifically, some authors have reported the involvement of an *alk* degradative system in the oxidation of internalized short-chain fragments of PE^20,21^. In R7 genome an *alkB* gene is present in a unique copy and it was previously studied for its involvement in medium-chain *n*-alkanes^55^. RT-qPCR expression experiments showed that R7 *alkB* gene is around 100-fold up-regulated under PE condition with respect to the reference condition which is in line with what was observed by Gravouil and coworkers^15^ for C208 strain.

Moreover, R7 transcriptome showed also an up-regulated gene encoding a cytochrome P450 (AII08421) similar to CYP124 of *Mycobacterium* spp., CYP125 of *Rhodococcus erythropolis* PR4 (RER_33720), and *Rhodococcus jostii* RHA1 (WP_009477746) with around 30% amino acid identity, indicating that R7 P450 belongs to CYP124 or CYP125 families. These enzymatic classes catalyze the side-chain cholesterol degradation or are activated in the presence of diesel oil, thus, involved in the biodegradation of aliphatic carbon chains^52–54^.

Overall, these preliminary suggestions indicated the possible involvement of R7 different oxidative systems in the degradation of internalized short-chain PE.

Finally, R7 PE mineralization can be achieved through the induction of genes for the β-oxidation pathway. Indeed, five up-regulated genes of R7 were identified as good candidates to accomplish the complete degradation of saturated fatty acids as observed by Gravouil and coworkers^15^.

Consistent with this hypothesis, among the overexpressed genes we looked for genes encoding transport systems. Diverse R7 genes encoding different membrane proteins (3 different genes), an integral membrane protein, a putative ATP/GTP-binding integral membrane protein, an ABC transporter ATP-binding protein, and a protein-export membrane protein SecD (TC 3.A.5.1.1) were activated during PE degradative process.

In order to confirm the ability of R7 to degrade PE and the consequent expression of specific genes, FTIR analysis was performed, and the results strongly suggested that PE growth condition compared to malate condition induces in R7 strain metabolic changes that involve mainly lipids and then proteins. Indeed, the most significant spectral differences between the two cases affect several absorption bands that are ascribable to CH_2_ groups of lipid hydrocarbon chains, which display a higher intensity in PE cell spectra. This result could indicate the presence of longer lipid hydrocarbon chains in the membranes compared to cells grown on malate, which affects the physical–chemical properties of cell lipids (for R7 cells grown on PE), as also supported by the spectral features of the C=O band between ~ 1760–1730. This absorption is responsive to changes in the carbonyl group environment, including hydrogen bonding and polarity^57^. Interestingly, R7 cells grown on the two different carbon sources displayed significant spectral differences also in this range. Furthermore, the FTIR result highlighted differences in the overall cell protein secondary structures in the two conditions. Particularly, R7 cells grown in the presence of PE showed a more intense *beta*-sheet band compared to R7cells grown on malate.

**Figure 7:**
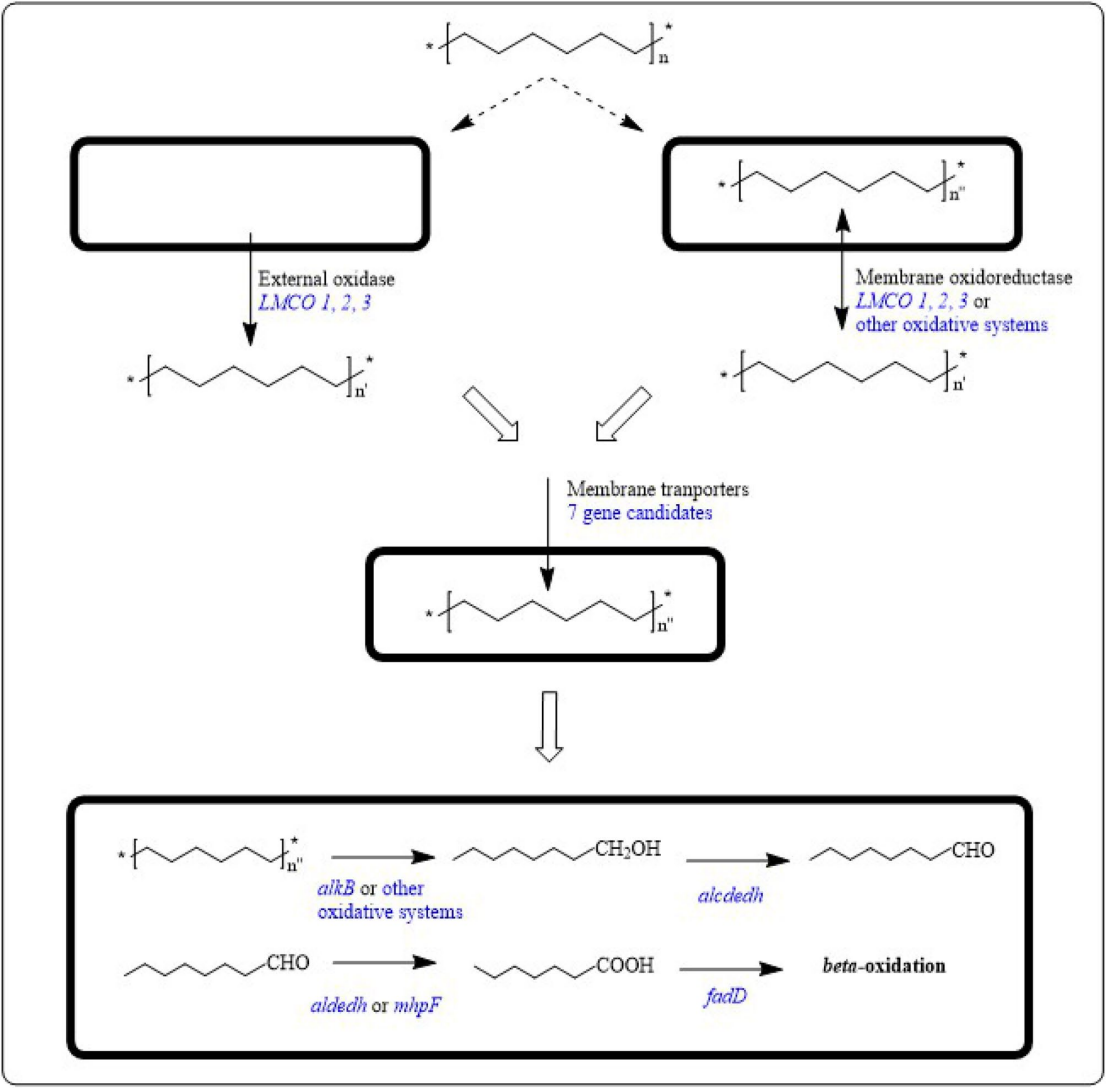
Proposed metabolic pathways of *R*. *opacus* R7 for polyethylene degradation. Blu genes represent candidate genes involved in PE metabolism: *LMCO 1*, *LMCO 2*, and *LMCO 3*, laccase-like multicopper oxidase; membrane oxidoreductase or other oxidative systems include *alkB* system; *alkB*, alkane monooxygenase; *alkB* or other oxidative systems include *cyp450*, cytochrome P450 hydroxylase. Membrane transporters include integral membrane proteins. *alcdedh*, alcohol dehydrogenase; *aldedh*, aldehyde dehydrogenase; *mhpF*, acetaldehyde dehydrogenase; *fadD*, long-chain-fatty-acid-CoA ligase. Names of enzyme categories reported in black color represent candidates for each metabolic step. Dashed arrows indicate hypothetical metabolic steps. Full white arrows indicate required metabolic steps.

In conclusion, this work provides evidence that *Rhodococcus opacus* R7 activates diverse oxidases and oxygenases to cope with PE as the only carbon and energy source. For the first time, the complete polyethylene degradative pathway is proposed. LMCO genes were up-regulated as the first step of oxidation upon PE outside exposure, indicating that this process is presumably performed on the cell membrane or on its interface with the extracellular environment. Consequently, more than one oxidoreductase system takes action on PE favoring the entrance of the smaller aliphatic fragments. Thus, R7 internally uses monooxygenases and hydroxylases to reduce alkane fragment length leading to the β-oxidation pathway (Fig. 7).

These findings are noteworthy, and they can be considered valuable to address the current plastic waste emergency in terms of a novel microorganism able to biodegrade polyethylene and above all new data on the genes involved in this degradation; as a matter of fact, the identification of these gene products poses the basis for diverse environmental and biotechnological applications contributing to plastic elimination. For example, they could be used as marker sequences for environmental biomonitoring as well as they can be useful as biotechnological catalysts for the biodegradation of polyethylene-based plastics.

## Supplementary Information

Supplementary Table S1.

Supplementary Table S2.

Supplementary Table S3.
