# Supplementary material for: Transcriptomic analysis of Rhodococcus opacus R7 grown on polyethylene by RNA-seq
Source: Sci Rep. 2021 Oct 29;11:21311. doi: 10.1038/s41598-021-00525-x (PMC8556283; doi:10.1038/s41598-021-00525-x)
Supplement: Supplementary file 2 — Supplementary Table S2. [file 41598_2021_525_MOESM2_ESM.docx]

**Table S2** List of oligonucleotides used for RT-qPCR analyses.

| **Oligonucleotide name** | **sequence (5’ - 3’)** |
| --- | --- |
| RT-16S-R7f | TCGTGAGATGTTGGGTTAAG |
| RT-16S-R7r | CCTCTGTACCGGCCATTGTAG |
| RTCuO5454f | TCGCAGGTACGCGCACTGCTG |
| RTCuO5454r | CGTCCATGTCGTTGCGGATGG |
| RTCuO9011f | GTCATCGGCCCCGACTCC |
| RTCuO9011r | GGTCGTTGTAGGCCCAGGTG |
| RTCuO9053f | GACTACGGCCGGGTCTCCCAG |
| RTCuO9053r | CACCTTCCTGGCAGCGCAGC |
| RT-alkB510-for | GACATGAGCCAGGCATTGAG |
| RT-alkB744-rev | CTGGCAAAGATCACTCTCGC |
