# Supplementary material for: Transcriptomic analysis of Rhodococcus opacus R7 grown on polyethylene by RNA-seq
Source: Sci Rep. 2021 Oct 29;11:21311. doi: 10.1038/s41598-021-00525-x (PMC8556283; doi:10.1038/s41598-021-00525-x)
Supplement: Supplementary file 3 — Supplementary Table S3. [file 41598_2021_525_MOESM3_ESM.docx]

**Table S3** - List of *R. opacus* R7 selected DEGs after RNA-seq and RT-qPCR analyses.

| **ID NCBI (aa)** | **Gene Name** | **LogFC** | **Location** | **Start** | **Stop** | **Length (bp)** | **Function** | **Subsystems** | **GO** |
| --- | --- | --- | --- | --- | --- | --- | --- | --- | --- |
| AII08809 | *LMCO1* | 19.5 | Chromosome | 6797144 | 6795660 | 1485 | Multicopper oxidase | Copper homeostasis | [GO:0005886] plasma membrane [GO:0005507] copper ion binding [GO:0042597] periplasmic space [GO:0004322] ferroxidase activity |
| [AII11185](https://www.ncbi.nlm.nih.gov/protein/AII11185.1?report=genbank&log$=protalign&blast_rank=1&RID=BHPP5GAF01R) | *LMCO2* | - | pPDG3 | 146277 | 147827 | 1551 | Multicopper oxidase | Copper homeostasis | [GO:0005886] plasma membrane [GO:0005507] copper ion binding [GO:0042597] periplasmic space [GO:0004322] ferroxidase activity |
| AII11221 | *LMCO3* | - | pPDG3 | 196575 | 195529 | 1047 | Multicopper oxidase | Copper homeostasis | [GO:0006807] nitrogen compound metabolic process [GO:0005507] copper ion binding [GO:0009279] cell outer membrane [GO:0050421] nitrite reductase (NO-forming) activity |
| AII08802 | *benA* | 19.4 | Chromosome | 6788743 | 6787403 | 1341 | Benzoate 1,2-dioxygenase alpha subunit (EC 1.14.12.10) | Aromatic dioxygenase mess, Benzoate degradation, Dioxygenases (EC 1.14.12.-) | [GO:0005506] iron ion binding [GO:0051537] 2 iron, 2 sulfur cluster binding [GO:0043640] benzoate catabolic process via hydroxylation [GO:0018623] benzoate 1,2-dioxygenase activity |
| AII09363 | *-* | 18.2 | Chromosome | 7399071 | 7398793 | 279 | Sarcosine oxidase delta subunit (EC 1.5.3.1) | Choline and Betaine Uptake and Betaine Biosynthesis, Glutamate-mediated methylamine utilization pathway, Niacin-Choline transport and metabolism, Sarcosine oxidase subunits | [GO:0005737] cytoplasm [GO:0046653] tetrahydrofolate metabolic process [GO:0008115] sarcosine oxidase activity |
| [AII08803](https://www.ncbi.nlm.nih.gov/protein/AII08803.1?report=genbank&log$=protalign&blast_rank=1&RID=BHRDWEGE01R) | *-* | 16.9 | Chromosome | 6789955 | 6788960 | 996 | 2-polyprenylphenol hydroxylase and related flavodoxin oxidoreductases / CDP-6-deoxy-delta-3,4-glucoseen reductase-like | Central meta-cleavage pathway of aromatic compound degradation | [GO:0046872] metal ion binding [GO:0009055] electron transfer activity [GO:0051537] 2 iron, 2 sulfur cluster binding [GO:0018662] phenol 2-monooxygenase activity [GO:0046191] aerobic phenol-containing compound catabolic process |
| AII03285 | *-* | 4.5 | Chromosome | 212707 | 210941 | 1767 | conserved hypothetical hydroxylase | - | [GO:0071949] FAD binding [GO:0046300] 2,4-dichlorophenoxyacetic acid catabolic process [GO:0018666] 2,4-dichlorophenol 6-monooxygenase activity |
| AII08421 | *cyp450* | 4.0 | Chromosome | 6361443 | 6360121 | 1323 | putative cytochrome P450 hydroxylase | Nitric oxide synthase | [GO:0005737] cytoplasm [GO:0005506] iron ion binding [GO:0016705] oxidoreductase activity, acting on paired donors, with incorporation or reduction of molecular oxygen [GO:0004497] monooxygenase activity [GO:0020037] heme binding |
| AII08632 | *alkB* | 2.9 | Chromosome | 6591488 | 6590256 | 1233 | Alkane-1 monooxygenase (EC 1.14.15.3) | - | [GO:0005886] plasma membrane [GO:0016021] integral component of membrane [GO:0046872] metal ion binding [GO:0006629] lipid metabolic process [GO:0018685] alkane 1-monooxygenase activity [GO:0052869] arachidonic acid omega-hydroxylase activity [GO:0043448] alkane catabolic process |
| AII10792 | *-* | 5.5 | pPDG2 | 15656 | 17419 | 1764 | Possible membrane protein | - | [GO:0003677] DNA binding [GO:0005524] ATP binding [GO:0016887] ATPase activity [GO:0019899] enzyme binding [GO:0005634] nucleus [GO:0032389] MutLalpha complex [GO:0006298] mismatch repair [GO:0042493] response to drug [GO:0032300] mismatch repair complex [GO:0030983] mismatched DNA binding |
| AII10609 | *-* | 3.0 | pPDG1 | 410211 | 411344 | 1134 | Integral membrane protein | - | [GO:0005886] plasma membrane [GO:0016021] integral component of membrane |
| AII04246 | *-* | 2.0 | Chromosome | 1379567 | 1378551 | 1017 | putative ATP/GTP-binding integral membrane protein | - | [GO:0005524] ATP binding [GO:0016887] ATPase activity [GO:0005737] cytoplasm [GO:0007049] cell cycle [GO:0051301] cell division [GO:0032153] cell division site |
| AII09203 | *-* | 1.3 | Chromosome | 7214400 | 7215494 | 1095 | Methionine ABC transporter ATP-binding protein | Methionine Biosynthesis, Methionine Degradation | [GO:0005618] cell wall [GO:0005886] plasma membrane [GO:0005524] ATP binding [GO:0016887] ATPase activity [GO:0070508] cholesterol import |
| AII04221 | *secD* | 2.3 | scaffold00001 | 1356701 | 1355031 | 1671 | Protein-export membrane protein SecD (TC 3.A.5.1.1) | - | [GO:0005618] cell wall [GO:0005886] plasma membrane [GO:0005887] integral component of plasma membrane [GO:0005829] cytosol [GO:0005576] extracellular region [GO:0015031] protein transport [GO:0006605] protein targeting [GO:0065002] intracellular protein transmembrane transport [GO:0015450] P-P-bond-hydrolysis-driven protein transmembrane transporter activity [GO:0052059] modulation by symbiont of defense-related host reactive oxygen species production [GO:0043952] protein transport by the Sec complex |
| [AII06740](https://www.ncbi.nlm.nih.gov/protein/AII06740.1?report=genbank&log$=protalign&blast_rank=1&RID=BHU7A1C001R) | *-* | 17.7 | scaffold00001 | 4330096 | 4329521 | 576 | FIG054221: Possible conserved alanine rich membrane protein | - |  |
| AII07038 | *-* | 18.7 | scaffold00001 | 4671268 | 4670363 | 906 | Possible conserved integral membrane protein | - | [GO:0016021] integral component of membrane [GO:0071555] cell wall organization [GO:0016765] transferase activity, transferring alkyl or aryl (other than methyl) groups [GO:0045227] capsule polysaccharide biosynthetic process |
| AII08419 | *alcdedh* | 4.8 | scaffold00001 | 6358831 | 6357725 | 1107 | benzyl alcohol dehydrogenase | - | [GO:0008270] zinc ion binding [GO:0019439] aromatic compound catabolic process [GO:0018456] aryl-alcohol dehydrogenase (NAD+) activity |
| AII08418 | *aldedh* | 4.4 | scaffold00001 | 6357688 | 6356252 | 1437 | Aldehyde dehydrogenase (EC 1.2.1.3) | Glycerolipid and Glycerophospholipid Metabolism in Bacteria, Methylglyoxal Metabolism, Methylglyoxal Metabolism, Pyruvate metabolism II: acetyl-CoA, acetogenesis from pyruvate | [GO:0051287] NAD binding [GO:0034832] geranial dehydrogenase activity [GO:0071310] cellular response to organic substance [GO:0043694] monoterpene catabolic process [GO:0016098] monoterpenoid metabolic process |
| AII03621 | *mhpF* | 17.7 | scaffold00001 | 633448 | 632477 | 972 | Acetaldehyde dehydrogenase, acetylating, (EC 1.2.1.10) in gene cluster for degradation of phenols, cresols, catechol | Biphenyl Degradation, Biphenyl Degradation, Central meta-cleavage pathway of aromatic compound degradation | [GO:0019439] aromatic compound catabolic process [GO:0051287] NAD binding [GO:0008774] acetaldehyde dehydrogenase (acetylating) activity |
| similar to WP_128639699 | *fadD* | 6.2 | scaffold00001 | 3180679 | 3182526 | 1848 | Long-chain-fatty-acid-CoA ligase (EC 6.2.1.3) | Biotin biosynthesis, Biotin synthesis cluster, Fatty acid metabolism cluster | [GO:0005524] ATP binding [GO:0046872] metal ion binding [GO:0003996] acyl-CoA ligase activity [GO:0102391] decanoate-CoA ligase activity [GO:0004467] long-chain fatty acid-CoA ligase activity |
| AII05967 | *hbd* | 4.1 | scaffold00001 | 3468667 | 3469515 | 849 | 3-hydroxybutyryl-CoA dehydrogenase (EC 1.1.1.157); 3-hydroxyacyl-CoA dehydrogenase (EC 1.1.1.35) | Acetyl-CoA fermentation to Butyrate, Acetyl-CoA fermentation to Butyrate, Butanol Biosynthesis, Butyrate metabolism cluster, Fatty acid metabolism cluster, Isoleucine degradation, Polyhydroxybutyrate metabolism, Polyhydroxybutyrate metabolism, Valine degradation | [GO:0005618] cell wall [GO:0005886] plasma membrane [GO:0005829] cytosol [GO:0003857] 3-hydroxyacyl-CoA dehydrogenase activity [GO:0008691] 3-hydroxybutyryl-CoA dehydrogenase activity [GO:0006635] fatty acid beta-oxidation [GO:0070403] NAD+ binding [GO:0030497] fatty acid elongation [GO:0019605] butyrate metabolic process [GO:0052572] response to host immune response |
